# The Leeds Evaluation of Efficacy of Detoxification Study (LEEDS) Prisons Project Study: protocol for a randomised controlled trial comparing methadone and buprenorphine for opiate detoxification

**DOI:** 10.1186/1745-6215-10-53

**Published:** 2009-07-14

**Authors:** Laura Sheard, Nat MJ Wright, Clive E Adams, Nicole Bound, Bruno Rushforth, Roger Hart, Charlotte NE Tompkins

**Affiliations:** 1 Leeds Primary Care Trust based at Leeds Institute for Health Sciences, 101 Clarendon Road, Leeds, LS2 9LJ, UK; 2 Faculty of Medicine and Health Sciences, University of Nottingham, Nottingham, NG7 2UH, UK; 3 Formerly HMP Leeds, 2 Gloucester Terrace, Armley, Leeds, LS12 2TJ, UK

## Abstract

**Background:**

In the United Kingdom (UK), there is an extensive market for the class 'A' drug heroin and many heroin users spend time in prison. People addicted to heroin often require prescribed medication when attempting to cease their drug use. The most commonly used detoxification agents in UK prisons are currently buprenorphine and methadone, both are recommended by national clinical guidelines. However, these agents have never been compared for opiate detoxification in the prison estate and there is a general paucity of research evaluating the most effective treatment for opiate detoxification in prisons. This study seeks to address this paucity by evaluating the most routinely used interventions amongst drug users within UK prisons.

**Methods/Design:**

This study uses randomised controlled trial methodology to compare the open use of buprenorphine and methadone for opiate detoxification, given in the context of routine care, within three UK prisons. Prisoners who are eligible and give informed consent will be entered into the trial. The primary outcome will be abstinence status eight days after detoxification, as determined by a urine test. Secondary outcomes will be recorded during the detoxification and then at one, three and six months post-detoxification.

**Trial registration:**

Current Controlled Trials ISRCTN58823759

## Background

In the United Kingdom (U.K), there is an extensive market for the sale of heroin, an illicit class 'A' drug. Precise figures of how many people use and are dependent on heroin are difficult to establish as there has never been a national prevalence survey [1]. However, a 2004 survey found that 67% of people receiving drug treatment (out of a population of 125 545) identified heroin as their main problem drug [2]. The link between crime and illicit drug use, specifically heroin use, is well recognised. In 2002, 63% of injecting drug users (IDUs) in contact with specialist drugs services in England and Wales reported having ever been in prison or a young offenders' institution [3]. Because of the drug's highly addictive properties, those addicted to illicit opiates such as heroin require medical help in reducing and stopping their use.

Previously, neither the evidence base [1] nor the UK national guidelines on the clinical management of drug misuse [4] stipulated a 'drug of choice' for opiate detoxification in the prison setting. In the absence of an established evidence base, a wide variety of agents have therefore been used, they include methadone, dihydrocodeine, buprenorphine, lofexidine and clonidine. Currently, methadone and buprenorphine are recommended by national clinical guidelines [5]. Prior to the mid 2000s, the most commonly used drug for opiate detoxification had been dihydrocodeine. Anecdotally, dihydrocodeine was used because of a reluctance to prescribe methadone following a small number of methadone related deaths in prisons. Dihydrocodeine is attractive to clinicians as it has a shorter half-life than methadone, and seems equally acceptable to users. There was a move away from prescribing dihydrocodeine because of its potential for diversion by prisoners into the prison shadow economy, buprenorphine has been increasingly prescribed. Buprenorphine – in the form of sub-lingual tablets – has the potential advantage of having a good safety profile, a higher rate of adherence and lower withdrawal severity when compared to methadone, lofexidine or clonidine [6-9]. Its introduction into the prison estate was in line with increasing prescribing in the community [10]. An evidence base exists in the community for the effectiveness of methadone in achieving detoxification [4]. In 2004, policy initiatives recommended increasing the provision of methadone programmes within the prison setting [11]. Consequently, methadone was re-introduced into the prison estate in accordance with current best practice guidelines for prescription and administration.

Despite the changes highlighted above regarding recommendations for first-line agents, few clinical trials conducted in the UK prison setting have evaluated medication for opiate detoxification. Whilst one study evaluated the withdrawal severity of methadone and lofexidine [12], the rates of completion were not sufficient to detect a statistically significant difference between the two medications. In 2004, the Leeds Evaluation of Efficacy of Detoxification Study (LEEDS) team conducted the first randomised controlled trial (RCT) comparing buprenorphine and dihydrocodeine for opiate detoxification in the community [13,14]. The research team then compared these two agents in a pilot trial in HMP (Her Majesty's Prison) Leeds [15], and recently submitted the results for publication. The LEEDS trial has since expanded to compare the open use of buprenorphine to methadone across healthcare in several prisons.

## Methods

### Design

LEEDS is a pragmatic open label randomised controlled trial.

Randomisation is by random block size, which CA administers centrally. He prepares opaque consecutively numbered envelopes. If a prisoner is both eligible and agreeable, the next envelope is opened and the intervention allocated. Randomisation controls for confounding variables (e.g. doctor-participant relationships, drugs worker-participant).

### Setting and Recruitment

The study is taking place in prison healthcare at HMP Leeds and HMP New Hall in West Yorkshire and HMP Durham in County Durham, England. All prisons are category B. (this refers to a prison which is high but not maximum security). HMP Leeds is a local prison, it accepts only adult male prisoners aged 21 and over from West Yorkshire. HMP New Hall, based in a rural location, is a local women's prison for adult females. HMP Durham accepts male prisoners from the North East of England. NB, RH and BR recruit participants from the medical reception area on first arrival into HMP Leeds, where prisoners are routinely offered a detoxification regime. Recruitment began in HMP Leeds in January 2006, HMP Durham in February 2007 and in HMP New Hall in January 2008. Data collection is ongoing.

### Sample size

In the situation of helping people detoxify from heroin, even a small advantage for an intervention could represent a worthwhile benefit. Therefore LEEDS has been planned to detect even a 15% difference in the proportion of opiate-free patients within the detoxification period i.e. percentage already abstinence 35% in one group and 50% in the other group. LEEDS expects to recruit 340 participants. A sample size of 340 people would yield at least an 80% chance (1 - β error or power) of detecting an absolute difference of 15% between the proportion of opiate-free patients in each group, at a two sided 5% level of significance (α error).

### Eligibility

#### Inclusion criteria

1. Male or female

2. 18 – 65 years old

3. Using illicit opiates as confirmed by a urine test taken at first assessment

4. Expressing a wish to detoxify through the standard monitored process and remain abstinent from opiates

5. Willing to give informed consent after receiving the participant information booklet

6. Remaining in custody for at least 28 days

#### Exclusion criteria

1. Contraindications to methadone or buprenorphine

2. Co-existing acute medical conditions requiring emergency admission for hospital care thus precluding detoxification in the prison setting

3. Currently undergoing detoxification from other addictive drugs whereby concurrent detoxification from opiates would not be clinically indicated

4. Previously been randomised into this trial

### Interventions

The treatment option will be concealed from both the participant and clinician/drugs worker at time of assessment. Both parties will remain blind to the selected treatment option until the envelope is opened.

1. Methadone, given openly, in the context of the standard prison doctor and drugs worker support.

2. Buprenorphine, given openly, in the context of the standard prison doctor and drugs worker support.

The reducing regimen of both methadone and buprenorphine (over less than 20 days) will be at the discretion of the prescribing doctor. However, the dose should not exceed this standard regime (Table 1).

**Table 1:** Detoxification schedules

**Day**	**Buprenorphine** **(mg)**	**Methadone** **(1 mg/1 ml mixture)**
1	8	30
2	8	30
3	8	30
4	8	30
5	8	30
6	6	25
7	6	25
8	4	22
9	4	22
10	4	20
11	3.6	20
12	3.6	18
13	3.2	16
14	2.8	14
15	2.4	12
16	2.0	10
17	1.6	8
18	1.2	6
19	0.8	4
20	0.4	2

### Consent

Consent for the participant to enter the trial is gained at first presentation to the prison doctor or drugs worker after confirmation of eligibility criteria. Written consent will be obtained after the participant has made an informed decision. Information will be provided to the participant using an information leaflet documenting the aims, objectives and necessity of the study. The study has been approved by the research ethics committee (MREC Northern and Yorkshire).

### Outcomes

#### Primary outcome

Abstinence from illicit opiates at eight days post detox as indicated by urine test. A person who does not finish the course of detoxification or refuses to give urine will be considered not abstinent. If participants are released before the date of the urine test then the primary outcome will be based on self report in the community. If self report date is unobtainable then they will be noted as "loss to follow up".

#### Secondary outcomes

During the period of detoxification

##### Adverse events

Clinicians will record any details of adverse events in the usual way by making an entry in the participants' records. The researchers based at the prisons inform LS immediately of any adverse events clearly resulting in clinically significant distress to study participants or of major concern to clinicians. Any serious or adverse events are then reported to regulatory authorities

##### Leaving the study early

Perceived reasons for withdrawal are recorded.

##### Inappropriate use of prescribed medication

Examples of this include storing, trading, swapping or selling of prescribed medication.

###### Overdose, self harm or suicide attempt

##### Service related outcomes

In-patient stays in prison healthcare will be recorded as will visits to a doctor, nurse or drugs worker

At one-month, three-month and six-month post detoxification

##### Abstinence status

Abstinence ascertained via urine test if the participant is still in HMP Leeds, Durham or New Hall, or has been transferred to another prison. If the participant has been released into the community then local community drugs service records are accessed.

All other outcomes as detailed above if the participant is still in the prison environment in which they were randomised.

The project team recognise the difficulty in tracking people with drug problems who have subsequently been released from prison across this period, particularly through the experience of conducting our pilot trial [15].

### Data collection

In HMP Leeds, NB, RH and BR collect baseline demographic data at randomisation. RH and NB collect the primary outcome and data at one, three months and six months after randomisation. Nursing staff and drugs workers collect data in the other centres. LS collates all data. There will be minimal additional contact with the participants. LEEDS is designed to avoid complicating the care provided to this group.

### Analysis

LS enters all data into an Excel spreadsheet. The analysis will be undertaken using Epi-Info software. The analysis of the primary outcomes will be of a 2 × 2 table (see Table 2). Dummy tables have been constructed for all secondary outcomes. These tables are designed to be rigid templates for the final write-up of the project, and to protect the researchers from bias, once the data are disclosed. Analysis will use relative risk tests for categorical data and unpaired t-tests for continuous data.

**Table 2:** Dummy table for abstinence from illicit heroin at final prescription as indicated by urine test

	Abstinence successful	Abstinence not successful	Totals
Buprenorphine	A	C	A+C

Methadone	B	D	B+D

Totals	A+B	C+D	A+B+C+D

## Discussion

As with our pilot trial in HMP Leeds [15], the information which the LEEDS project team aim to gain from this current trial is twofold. Firstly, once data collection is complete, we hope to show either a statistically significant difference between the efficacy of the two detoxification agents or alternatively, that no statistically significant difference exists (shown by p = > 0.05). We expect that the results will influence national prison healthcare policy and prescribing guidelines if the trial demonstrates one detoxification agent is significantly more efficacious than another.

Information about the methodological practicalities of conducting a multi centre, randomised controlled trial in the prison setting will be valuable. The research team have previously described the practicalities of conducting a detoxification RCT in primary care [13,14]. To our knowledge, however, only one British prison RCT comparing detoxification agents exists [12] aside from our prison pilot trial [15]. Therefore, conducting this current trial will provide new knowledge about the feasibility, practicality and day to day groundwork and management involved in running a trial in multiple prisons. This information may be important for future research teams. If successful, the trial will demonstrate the feasibility of multi centre trials within this environment.

Guidelines for substance misuse treatment in prison are not grounded on an established evidence base as there are very few trials conducted in this setting. Consequently, the project team envisage that this trial will provide valuable data about the efficacy of two routinely used detoxification agents which have rarely been studied within the prison environment.

## Approvals process

The extensive process of obtaining all necessary approvals for this trial and the issues surrounding this have been fully discussed in Sheard et al (2006) [16]. Research Governance approval was granted by Bradford South and West Primary Care Trust (PCT) on 2^nd ^December 2004. Research Ethics approval was granted from Multi Centre Research Ethics Committee (MREC) Northern and Yorkshire on 5^th ^July 2005.

The approval of the sponsor was gained on 28^th ^June 2005. A Clinical Trials Authority Certificate was issued from the Medicines and Healthcare Products Regulatory Authority on 28^th ^September 2005. The International Standard Randomised Controlled Trials number was granted on 15^th ^September 2005 (but backdated to 16 August 2005). Research governance for HMP Durham as an additional centre was granted by County Durham Primary Care Trust on 23^rd ^October 2006.

## Competing interests

The authors declare that they have no competing interests.

## Authors' contributions

NW and CA designed the study and offered project supervision. NW, CA and LS drafted the proposal and funding bid application. NW is chief investigator. CA was previously chief investigator and centrally manages the randomisation process. LS co-ordinates and manages the project and collects data. NB, RH and BR randomised participants into the trial. NB and RH collected data. All authors drafted and/or revised the manuscript.
